# Oral shedding of human herpesviruses in patients undergoing radiotherapy/chemotherapy for head and neck squamous cell carcinoma is not affected by xerostomia

**DOI:** 10.1080/20002297.2018.1476643

**Published:** 2018-05-28

**Authors:** Michelle Palmieri, Mariana Ornaghi, Victor Adriano de Oliveira Martins, Luciana Correa, Thais Bianca Brandao, Ana Carolina do Prado Ribeiro, Laura Masami Sumita, Tania Regina Tozetto-Mendoza, Claudio Sergio Pannuti, Paulo Henrique Braz-Silva

**Affiliations:** a Division of Pathology, Department of Stomatology, School of Dentistry, University of Sao Paulo, Sao Paulo, Brazil; b Division of Dentistry, Instituto do Cancer do Estado de Sao Paulo Octavio Frias de Oliveira, Sao Paulo, Brazil; c Laboratory of Virology, Institute of Tropical Medicine of Sao Paulo, University of Sao Paulo, Sao Paulo, Brazil

**Keywords:** Human herpesviruses, radiotherapy, chemotherapy, squamous cell carcinoma, xerostomia

## Abstract

**Background**: Xerostomia is a very relevant and frequent complication of radiotherapy, causing the irradiated oral mucosa to be affected by bacterial, fungal and viral infections.

**Objective**: The objective of this study was to evaluate a possible relationship between oral shedding of human herpesviruses and xerostomia in patients with squamous cell carcinoma of head and neck submitted to radio/chemotherapy.

**Methods**: In this study, oral rinse samples were collected weekly from 20 patients during radiotherapy. The samples were submitted to PCR and enzymatic digestion for detection of human herpesviruses. Xerostomia was evaluated according to the Seminars in Radiation Oncology criteria.

**Results**: There was a higher frequency of grade 1 xerostomia (51.4%), observed first in the 1st week of radiotherapy. In the 4th week of radiotherapy, all patients presented some degree of xerostomia. Analysis of herpesviruses showed oral shedding of EBV, HHV-6 and HHV-7 in all weeks. Considering all the periods, the highest frequency was in patients with EBV excretion (55.0%), which was significantly higher than that of other viruses.

**Conclusion**: We observed that oral shedding of herpesviruses was not affected by xerostomia as there was a progression in their excretion, even with the evolution of xerostomia. This suggested that there is a local replication in the oral cavity that is not completely dependent of salivary excretion.

## Introduction

Radiotherapy (RT) is an antineoplastic treatment capable of providing high growth control rates for stage 1 (80%) and stage 2 (60%–70%) tumours while preserving important anatomical structures [1,2]. Despite its effectiveness, the RT for head and neck tumours is accompanied by a number of complications arising from the involvement of radiosensitive tissues located close to the tumour [3–5].

Xerostomia is a relevant complication of RT caused by possible destruction of the glandular parenchyma, thus diminishing the production of saliva. The reduction of salivary flow causes discomfort to patients, and in the most severe cases, interferes with chewing, swallowing and speech. Clinical symptoms such as oral mucosal burning, dry lips, lip ridges, altered tongue surface, difficult adaptation of dental prosthesis, as well as denture stomatitis, are commonly reported by patients with xerostomia [6].

A study by Caielli et al. demonstrated that, in association with the reduction of salivary flow, xerostomia can also be characterised by salivary thickening and pH change during the treatment weeks. According to the authors, serous acini are the first to undergo alterations resulting from radiotherapy, followed by mucous membranes and duct cells. There is a consensus that changes in the salivary flow will happen when salivary glands receive more than 50 Gy of radiation, and below that, they can be transitory and limited [7]. Xerostomia is commonly reported by patients after the first week of RT, and an increase in its severity is observed along the weeks of RT [8]. The sensation of dry mouth may extend for long periods after the end of RT [9].

The irradiated oral mucosa can be affected by bacterial, fungal and viral infections as described by different authors, exacerbating the manifestation of RT adverse effects [10–13]. The association between viral infections and oral complications from RT are poorly reported in the literature, and the existing results are contradictory. Some authors suggest that RT may cause a transient state of immunosuppression, thus contributing to reactivation of herpesviruses [14]. Until this moment, there have been few studies in the literature relating oral shedding of human herpesviruses to xerostomia in patients undergoing RT associated with chemotherapy [15–17].

The aim of this study was to evaluate the oral shedding of human herpesviruses, namely, HSV-1 and 2 (herpes simplex viruses 1 and 2), EBV (Epstein-Barr virus), CMV (cytomegalovirus), VZV (varicella-zoster virus), HHV-6, HHV-7 and HHV-8 (human herpesviruses 6, 7 and 8), and their possible relationship with xerostomia in patients undergoing RT associated with chemotherapy for squamous cell carcinoma in the head and neck region.

## Material and methods

This study was approved by the Research Ethics Committee of the University of São Paulo School of Medicine according to protocol number 910.924 (CAAE 37922114.9.0000.0065).

In our study, we have analysed 158 oral rinse samples which were collected weekly from 20 patients admitted to the Division of Dentistry of the São Paulo Institute of Cancer (ICESP) for RT for squamous cell carcinoma in head and neck. Patients were selected if they met the eligibility criteria as follows: diagnosis of head and neck squamous cell carcinoma, treatment protocol with three-dimensional RT (3D-RT) with a total dose of 60 Gy associated with chemotherapy with cisplatin 100 mg/m^2^, no surgery for tumour resection, dental treatment before starting RT, oral hygiene orientation and low-laser level therapy during RT to prevent and treat oral mucositis. The medical history of every patient was obtained through records for collection of data on gender, age, tumour location and the TNM classification of malignant tumours.

The treatment consisted of 3D-RT with a total dose of 60 Gy, divided into 30 fractions daily for five consecutive days a week for approximately six weeks. Chemotherapy was performed with cisplatin 100 mg/m^2^ in three cycles every 21 days (days 1, 22 and 43), starting on day one of RT.

The clinical evaluation of xerostomia was conducted by the same dentist, beginning at the screening visit and then throughout the six weeks of RT (after each RT session) and in the follow-up visit (1 month after the end of RT treatment). The evaluation was performed according to criteria set by the Seminars in Radiation Oncology (Table 1) [18].

**Table 1.** Classification of xerostomia (Seminars in Radiation Oncology, ref 18).

Grade 0	Grade 1	Grade 2	Grade 3
None	Discreet symptoms,	Symptoms,	Severe symptoms,
	Without dietary changes	With significant dietary changes	Interference in diet, sleeping, speaking and other activities
		(necessity of liquid to swallow)	Tube feeding

The serology for human herpesviruses was conducted at the Laboratory of Virology, Institute of Tropical Medicine of Sao Paulo, by using ELISA kits (AbCam®, Cambridge, UK) for HSV-1, HSV-2, CMV, VZV and EBV on three different occasions: screening visit, last RT session and 30 days after the end of RT.

Oral rinse samples were collected from patients by instructing them to perform a mouthwash with 5 ml of distilled water for 30 s. The samples were stored at −80°C after being simultaneously collected during evaluation of xerostomia, i.e. at the screening visit, in the six weeks of RT and on the follow-up visit.

From these samples, according to a previous work of ours [13,19], we extracted DNA by using a semi-automatic method (NucliSENS® easyMAG® system, BioMérieux, Durham, NC) before amplification with PCR by using two sets of primers (i.e. HSVP1/P2 and VZVP1/P2) which correspond to a DNA sequence alignment from eight human herpesvirus described by Johnson [20] (Table 2). The first set, HSVP1/P2, amplifies the subtypes HSV-1, HSV-2, EBV, CMV and HHV-8 and the second set, VZVP1/P2, amplifies the subtypes VZV, HHV-6 and HHV-7. The positive samples were submitted to enzymatic digestion with BamHI and BstUI restriction enzymes (New England Biolabs) for specific determination of each one of the eight herpesviruses, also described in a previous work of ours [13,19].

**Table 2.** Sequence alignment for herpesviruses DNA.

Primers	
HSVP1	5ʹ-GTGGTGGACTTTGCCAGCCTGTACCC-3’
HSVP2	5ʹ-TAAACATGGAGTCCGTGTCGCCGTAGATGA-3’
VZVP1	5ʹ-GTCGTGTTTGATTTTCAAAGTTTATATCC-3’
VZVP2	5ʹ-ATAAACACACAATCCGTATCACCATAAATAACCT-3’

All the patients were daily submitted to prophylactic low-level laser therapy after each RT session, starting on the first day, as described previously [13]. In addition to laser therapy, the patients were instructed about oral hygiene and also received artificial saliva and analgesic medication when necessary.

The results were presented in absolute and relative frequencies. For comparison of the frequencies of xerostomia grades in relation to the RT time points, we have used a chi-square test. McNemar´s test was applied for a paired analysis in relation to virus type at each time point. To compare the frequency of negative cases of herpesviruses at the moment of screening within the 1-month follow-up, we used Fisher´s Exact test. In order to assess whether there was a correlation between presence/grade of xerostomia and oral shedding of different herpesviruses, we have used Spearman’s test. The statistical software used was Biostat 5.0® (Belém, Pará, Brazil) at a significance level of 5%.

## Results

Fifty patients diagnosed for squamous cell carcinoma of the head and neck undergoing RT combined with chemotherapy were evaluated. Only 25 patients (50%) started RT, whereas the others were excluded for different reasons, namely: changes or suspension in the RT protocol, patients moving to other cities, patients refusing to participate in the study during sample collection and death. Five patients did not complete the RT, either because of treatment suspension due to toxicity (three patients) or because of death (two patients). Therefore, 20 patients completed the RT and met the eligibility criteria of our study. The general characteristics of these patients were: 19 males and one female, age between 39 and 72 years old (mean = 54.4 years). The tumour sites were: six in the oropharynx and soft palate, four in the oropharynx and base of the tongue, four in the base of the tongue, two in the floor of the mouth, one in the oropharynx and retromolar trigone, one in the retromolar trigone, one in the gums and one in the jugal mucosa. In relation to the TNM classification, we have observed three patients rated as T4N3M0, 10 as T4N2M0, two as T4N2M0, one as T3N0M0, one as T2N2M0, two as T2N0M0 and one as T1N1M0. All these characteristics were also described in a previous published work of our group [13].

## Xerostomia

Grade 1 of xerostomia had the highest frequency (51.4%), which was statistically different compared to grade 2 (21%; chi-square test, *p* < 0.001) and grade 3 (3.6%, chi-square test, *p* < 0.001), by analysing the frequencies of xerostomia observed during and one month post-RT.

**Figure 1.**
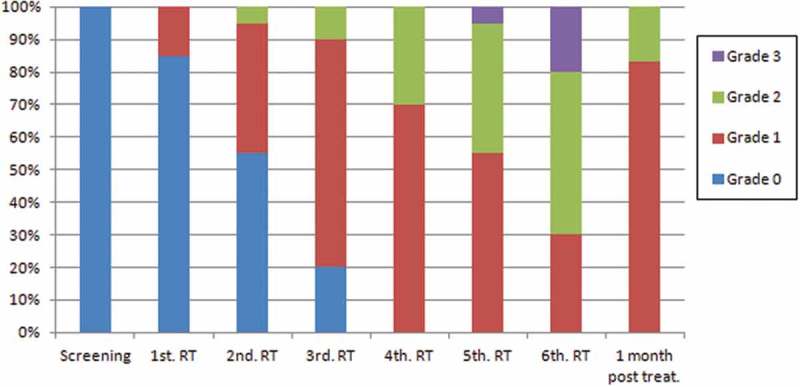
Degrees of xerostomia during the weeks of RT.

In the fourth week of RT, all patients presented some degree of xerostomia, but it was in the sixth week that the most severe degrees were observed (Figure 1).

Considering the degrees of xerostomia which cause dietary changes (grades 2 e 3), a significantly higher frequency of these xerostomia grades was observed in the last 3 weeks of RT (Figure 1) (chi-square test, *p* < 0.001).

In the follow-up visit (i.e. one month after the end of RT), 10 patients (55%) presented a decrease in the xerostomia degree, but without complete remission. Fifteen patients (83.3%) presented grade 1 and three patients (16.0%) grade 2 xerostomia (Figure 1).

## Human herpesviruses

### Serology

By analysing the IgG serology of these patients, which corresponds to previous contact with virus, it was possible to observe that the totality of patients (20 patients) presented IgG-antibodies to HSV1-HSV2, EBV and VZV in all three study periods: the screening visit, the last RT session and the follow-up visit. Only one patient was IgG negative for CMV in the three analysed periods.

Considering the IgM serology, which corresponds to recent viral contact or possible viral reactivation, one patient showed IgM-antibodies to HSV1-HSV2 and another one IgM-antibodies to CMV in all three study periods. Another patient presented IgM-antibodies to CMV detection in the last period, that is, 1 month after the end of RT, thus indicating a possible reactivation of this virus.

### Oral shedding of human herpesviruses

By analysing the oral shedding results for human herpesvirus in saliva, the results showed 18 patients (90%) with oral shedding of EBV, 14 (70%) with HHV-7, seven (35%) with HSV-1 and three (15%) with HHV-6. Possible co-detections, with 15 patients (75%) presenting shedding of two or more herpesviruses, were also observed. Four patients (20%) presented oral shedding of EBV only and one (5%) presented negative results for all human herpesviruses. By analysing all the patients together, the EBV oral shedding frequency was significantly higher compared to that of other herpesviruses (chi-square test, *p* < 0.001).

**Figure 2.**
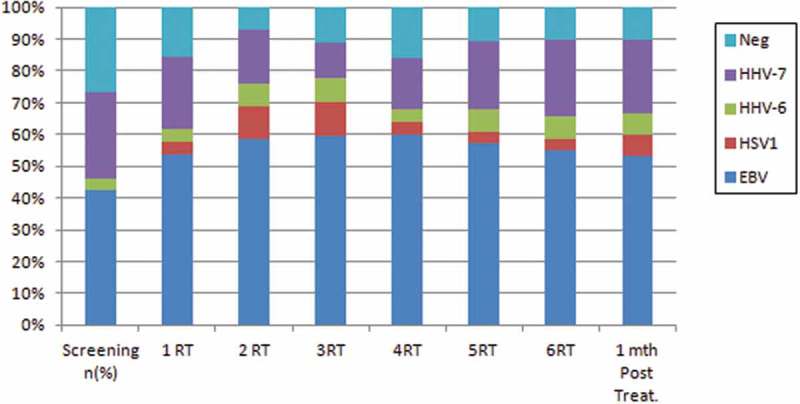
Frequency of oral shedding of human herpesviruses in the screening, along the weeks of RT, and one month after RT.

Considering the oral shedding of human herpesviruses and the weeks of RT, the results showed oral shedding of EBV, HHV-6 and HHV-7 in all weeks of RT (Figure 2). The oral shedding of HSV-1 was not observed in the screening visit (Figure 2). Table 1 shows the virus expression for each patient in accordance with the time points. The frequency of patients positive for EBV increased mainly from the second week, but the difference of this frequency in relation to the baseline (‘before RT’) was not significant (McNemar´s test, *p *= 0.070). The expression of the other virus types exhibited a great variance in the time points, but without significant differences in relation to the baseline (Table 3).

**Table 3.** Oral shedding for herpesviruses EBV, HHV-7, HSV-1 and HHV-6 in each patient in relation to the period before radiotherapy (RT) and each week during RT.

Blank spaces indicate negative cases. EB = EBV; H7 = HHV-7; H1 = HSV-1; H6 = HHV-6. *P* value for McNemar´s test, comparing ‘before RT’ with each week. Significant when *p *< 0.05

Analysis of the weeks of RT showed an increase in the number of patients with EBV oral shedding (85%) in the second week (Figure 2). There was no statistically significant difference in the EBV oral shedding frequencies observed in the first three weeks and those observed in the last three weeks of RT.

During the RT, negative samples for herpesvirus oral shedding were observed in all weeks (Figure 2 and Table 3). In the screening visit, a higher number of patients were observed (seven patients, 35%). A statistically significant difference was found between the frequency of negative cases in the screening visit and that of the follow-up visit (1 month after RT) as there was a decrease in the number of negative cases (Figure 2) (11%; Fisher’s Exact test, *p *= 0.043).

### Oral shedding of human herpesviruses and xerostomia

**Figure 3.**
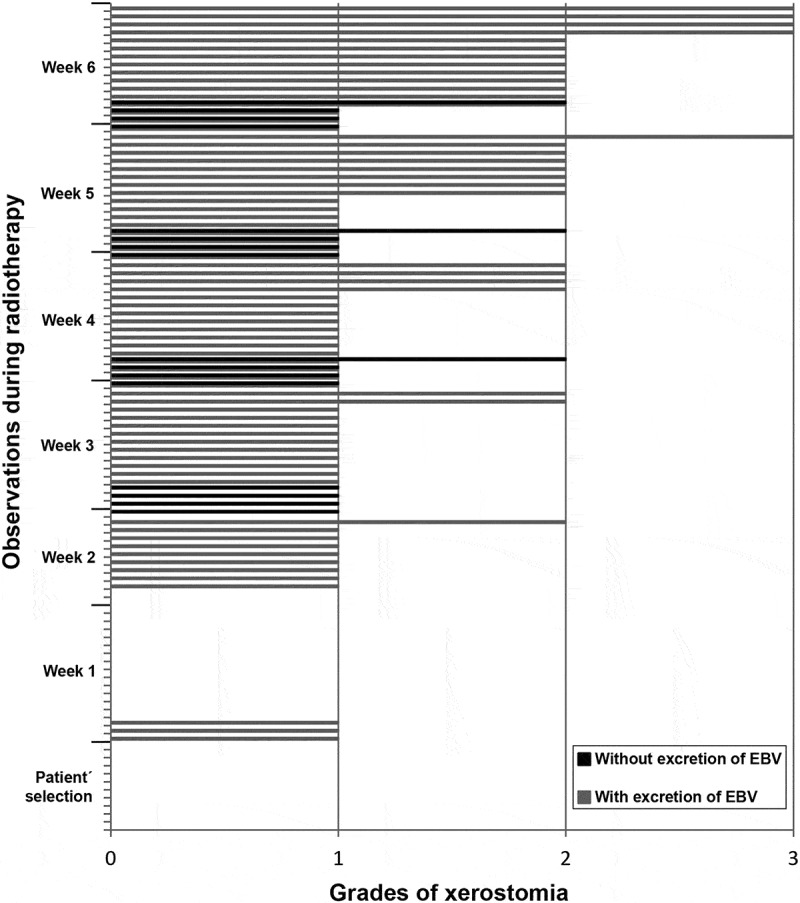
Frequency of EBV oral shedding in accordance with degrees of xerostomia observed in the screening and in each week of RT.

A correlation analysis between xerostomia and frequency of oral shedding of different viruses was performed. As for EBV, no significant correlation was found between frequency of xerostomia and excretion of EBV, not even when the most severe degrees of xerostomia (grade ≥ 2) were considered. Figure 3 shows the correlation between frequency of xerostomia and EBV excretion. It is noted that, in the majority of the cases, patients with and without excretion of EBV exhibited grade 1 xerostomia.

**Figure 4.**
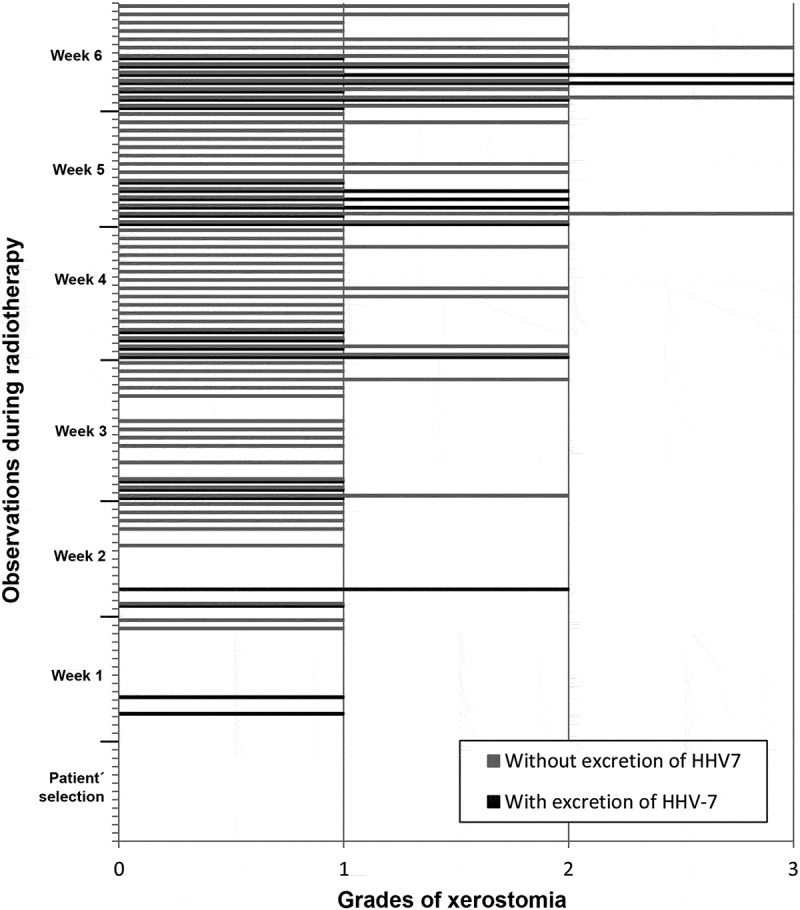
Frequency of HHV-7 oral shedding in accordance with degrees of xerostomia observed in the screening and in each week of RT.

**Figure 5.**
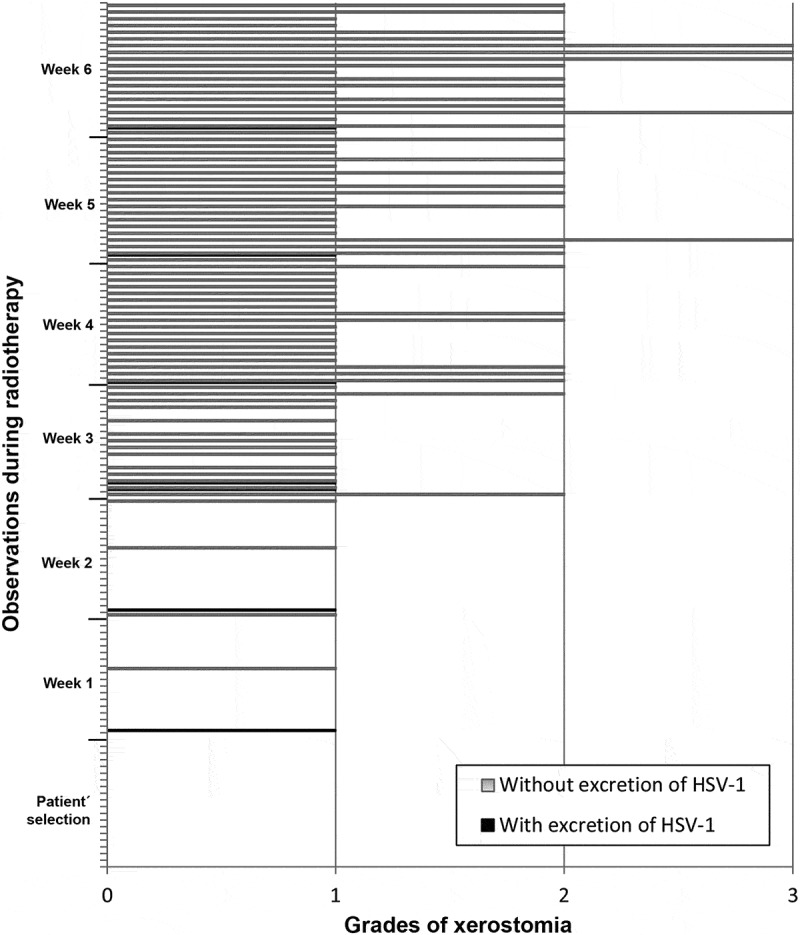
Frequency of HSV-1 oral shedding in accordance with degrees of xerostomia observed in the screening and in each week of RT.

**Figure 6.**
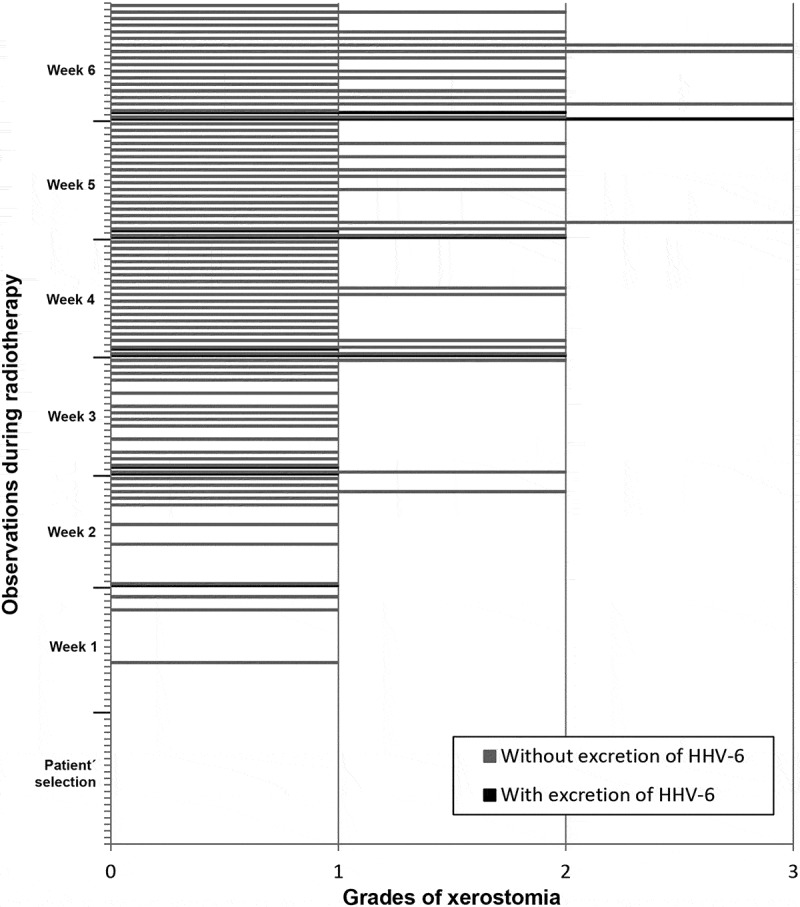
Frequency of HHV-6 oral shedding in accordance with degrees of xerostomia observed in the screening and in each week of RT.

In the analysis of the correlations between excretion of HHV-7, HSV-1 and HHV-6 and presence/degree of xerostomia, there was no significant relationship. Figures 4, 5 and 6 show the correlation between frequency of xerostomia and excretion of these viruses. The low number of cases in which HHV-7, HSV-1 and HHV-6 were excreted in association with a certain degree of xerostomia probably did not allow us to determine any correlation between these two variables.

By analysing the degree of xerostomia and presence of virus co-detection, we did not found any significant correlation between the xerostomia grade ≥ 2 and presence of herpesvirus co-detection along the RT weeks.

## Discussion

The RT used as a treatment for squamous cell carcinoma of the head and neck is widely known, as well as its oral complications. Patients undergoing RT may develop dysphagia, xerostomia, dysgeusia, mucositis and opportunistic infections (bacterial, fungal and viral) during treatment [12,21–23].

Different authors report a variety of oral complications resulting from radiotherapy. Among them, a systematic review by Trotti et al. showed that xerostomia was frequently found [24]. In the study by Cardoso et al. the authors observed that 100% of the patients irradiated with doses superior or equal to 60 Gy presented xerostomia [25], which was also observed in our study as all patients presented some degree of xerostomia. According to studies by Mosel et al. and Trotti et al. the sensation of dry mouth is usually observed in the third or fourth session of radiotherapy due to the lower secretion of saliva by the glands involved in the radiation field [12,24]. This dry mouth sensation impairs greatly the quality of life of the patients, interfering not only with chewing and swallowing, but also with speech, which limits their social life. In our study, we have observed the first reports of xerostomia in the first week of RT, with three patients (15%) presenting xerostomia grade 1. The authors also reported that xerostomia can be transient or permanent, depending on the irradiated field [12,24,25]. In our study, despite the short period of control after RT, 100% of the patients still had some degree of xerostomia in the follow-up visit, that is, one month after RT. Moreover, all the patients presented some degree of xerostomia in the fourth week of RT which persisted until the follow-up visit, meaning a possible permanent damage to the parenchyma of the salivary glands.

According to studies by Nicolatou-Galitis et al. and Correia et al. the great difficulty in comparing studies on xerostomia induced by head and neck RT and its possible correlation with HSV-1 is the lack of standardised samples. This may happen due to inclusion of different tumour types, patients with different protocol treatments for RT and/or chemotherapy, collection of oral rinsing samples performed at distinct moments, and lack of serological data [16,17,26].

In the present study, all the patients included were diagnosed with squamous cell carcinoma of head and neck and whose treatment protocol was RT, at a total dose of 60 Gy, associated with chemotherapy (Cisplatin 100 mg/m^2^). They had been also submitted to dental treatment before starting RT and chemotherapy. All the patients were treated with preventive and curative laser therapy for oral mucositis. The differential factor of our work was the weekly collection of oral rinsing samples during the whole period of RT, thus enabling a profile of the salivary excretion to be traced for different herpesviruses over time.

Our results have shown that five patients (25%) were positive for HSV-1 throughout the RT and two patients (10%) in the follow-up visit (1 month after treatment), with a rate of excretion of 35%. Of the total of patients followed up, none of them presented positive sample for HSV-1 before the beginning of RT treatment. However, there was no correlation between HSV-1 oral shedding and onset or worsening of xerostomia as well as no correlation with RT times either. A possible hypothesis for this lack of correlation with the degrees of xerostomia may be the control of the oral excretion of HSV-1 by the application of low-level laser, with similar results to the control of herpes labialis lesions [27–33].

It is known that HHV-6 and HHV-7 herpesviruses can be detected in healthy individuals who are asymptomatic [34] as exanthema subitum can be caused by a primary infection by HHV-6 and, less frequently, by HHV-7 [35,36]. The rate of oral excretion of HHV-6 in the healthy population is low (i.e. about 10%), while oral shedding rates of HHV-7 can vary from 12.6% to 90% depending on the population group [34,37]. HHV-6 and HHV-7 can be observed not only in healthy patients, but also in immunocompromised ones. A study by Pinheiro et al. evaluated the oral desquamation in children with positive HIV status, reporting that 68% of them presented HHV-6 and 18% presented HHV-7 in the oral cavity [36]. HHV-6 oral shedding seems to be higher and continuous in children compared to adults [38]. In our study, HHV-7 was the virus with the second highest frequency of oral shedding, responding for 70% of the positive samples (*p* = 0.001), whereas HHV-6 presented 15% of the oral shedding frequency. There was no statistical difference between HHV-6 and HSV-1 as well as no correlation between these viruses and xerostomia degrees or RT times.

In our study, EBV was the virus with the highest frequency of excretion during the study period, with 90% of the patients presenting positive sample. There was statistically significant difference compared to all other excreted viruses (*p* < 0.001). Ambinder et al. and Van der Beek et al. stated that 25%–98% of the saliva samples of immunosuppressed patients were positive for EBV as a result of chemotherapy [14,39]. According to the authors, all cancer patients presented some degree of immunosuppression, which may explain the higher rates of EBV excretion. It is widely reported in the literature that oncological patients present immunosuppression at different degrees, which may explain the higher rates of EBV excretion, as previously observed in other immunosuppressed groups for several reasons [19,40].

In another study by Pasoto et al. the authors analysed serum samples from 100 patients with Sjögren’s syndrome, a chronic autoimmune disease affecting exocrine glands (salivary and lachrymal) and which leads to xerostomia and keratitis sicca. As a result, they could assess the frequency of EBV antibodies in Sjögren’s patients, which were higher compared to healthy individuals. The authors concluded that EBV virus in the salivary glands, in its latent form, can occasionally resume its replicating form and become sub-clinically reactivated, suggesting that it could be a potentially aetiological candidate for triggering autoimmune diseases [41].

In our study, we have sought to evaluate whether the decrease in salivary excretion leads to a decrease in the oral shedding of human herpesviruses in the oral cavity. Our results showed that the xerostomia caused by RT does not affect the excretion of human herpesviruses, since it was possible to observe an increase in oral shedding of these herpesviruses in the oral cavity, even in the weeks when xerostomia was more severe. A hypothesis for this fact is that RT may have caused an alteration in the inflammatory microenvironment of the oral mucosa, favouring a possible local replication of herpesviruses in the oral cavity and so increasing the presence of them in this region.

In conclusion, the oral shedding of human herpesviruses is not affected by the presence of xerostomia because there was a progression in relation to the excretion of herpesviruses even with evolution of xerostomia, suggesting a local viral replication in the oral cavity without being completely dependent of salivary excretion.
