# Correction

**DOI:** 10.1080/20002297.2021.1890442

**Published:** 2021-03-29

**Article title**: Oral shedding of human herpesviruses in patients undergoing radiotherapy/chemotherapy for head and neck squamous cell carcinoma is not affected by xerostomia

**Authors**: Palmieri M, Ornaghi M, Martins VAO, Corrêa L, Brandao TB, Ribeiro ACP, Sumita LM, Tozetto-Mendoza TR, Pannuti CS and Braz-Silva P.

**Journal**: *Journal of Oral Microbiology*

**Bibliometrics**: Volume 10, Number 1, pages 1–9

**DOI**: 10.1080/20002297.2018.1476643

The above article was temporarily published incorrectly in issue 12-01 and that it has now been correctly placed within issue 10-01.

